# Thanks to our reviewers*, The Plant Genome*, 2020

**DOI:** 10.1002/tpg2.20108

**Published:** 2021-05-11

It is a great pleasure to thank the volunteer reviewers, without whom we could not publish *The Plant Genome*. Despite the demands of their everyday duties, the individuals listed below took time to assist the Editorial Board by reviewing one or more manuscripts in 2020. They demonstrate professionalism in the best sense. For their anonymous, unpaid efforts, all CSSA members owe them thanks. Every effort was made to compile an accurate list from the records kept by Editorial Board members. If we have omitted a name, we extend our apologies as well as our thanks.

Adhikari, Laxman, Manhattan, KS, USA

Akdemir, Deniz

Anderson, James, St. Paul, MN, USA

Bajgain, Prabin, Minneapolis, MN, USA

Bandillo, Nonoy

Batista, Lorena, Edinburgh, United Kingdom of Great Britain and Northern Ireland

Benhamed, Moussa

Bernardo, Rex, Saint Paul, MN, USA

Bhavani, Sridhar, Mexico DF, Mexico

Blas, Raul

Boualem, Adnane

Bradbury, Peter, Ithaca, NY, USA

Brar, Gurcharn, Vancouver, British Columbia, Canada

Buitink, Julia

Burt, Andrew, Ottawa, Ontario, Canada

Calert, Megan, Manhattan, KS, USA

Cameron, John, Davis, CA, USA

Campbell, Malachy, Blacksburg, VA, USA

Cao, Moju

Cao, Ke, Zhengzhou, China

Carter, Arron, Pullman, WA, USA

Castro, Patricia

Cavagnaro, Pablo F., USA

Chang, Yuxiao, Shenzhen, Guangdong, China

Chen, Lailiang

Chen, Charles, Stillwater, OK, USA

Chen, Liqing

Cichy, Karen, East Lansing, MI, USA

Clarke, John, Saskatoon, Canada

Clavijo, Bernardo

Cozzolino, Daniel

Crain, Jared, Manhattan, KS, USA

Curtin, Shaun

Curtin, Shaun, Saint Paul, MN, USA

Dawson, Julie, Madison, WI, USA

de Guzman, Christian

Dell'Acqua, Matteo

Deng, Zhiying, Taian, Shandong, China

Diao, Xianmin, Beijing, China

Diepenbrock, Christine, Davis, CA, USA

Edwards, Jacqueline, Bundoora, Victoria, Australia

Fan, Chuanzhu

Fellers, John, Manhattan, KS, USA

Flint‐Garcia, Sherry, Columbia, MO, USA

Friebe, Bernd, Manhattan, KS, USA

Friedt, Wolfgang

Funnell‐Harris, Deanna

Gemmer, Mathias, Halle, Germany

Givnish, Thomas, Madison, WI, USA

Goel, Shailendra, Delhi, India

Gomez, Francisco, East Lansing, MI, USA

Gouache, David, Pessac, France

Gowda, Manje, Nairobi, Kenya

Graham, Benjamin

Grover, Corrinne, Ames, IA, USA

Gu, Lianfeng

Guo, Tingting, Ames, IA, USA

Haile, Teketel, Saskatoon, Saskatchewan, Canada

Han, Dejun, Yangling, China

Hancock, Nathan

Hernandez Vasquez, Javier, Corvallis, OR, USA

Herniter, Ira, New Brunswick, NJ, USA

Hibberd, Julian

Hiebert, Colin, Winnipeg, Manitoba, Canada

Himmelbauer, Heinz, Wien, Austria

Ho, Julie, Davis, CA, USA

Holliday, Jason, Blacksburg, VA, USA

Hoshikawa, Ken, Tsukuba, Ibaraki, Japan

Howard, Reka, Lincoln, NE, USA

Hoyos‐Villegas, Valerio, Ste Anne de Bellevue, Quebec, Canada

Hufford, Matt

Huggins, Trevis, Stuttgart, AR, USA

Hyten, David, Lincoln, NE, USA

Iorizzo, Massimo, Kannapolis, NC, USA

Jarquin, Diego, Lincoln, NE, USA

Jayakodi, Murukarthick, Stadt Seeland, Germany

Jiang, Yong, Stadt Seeland, Germany

Jiao, Yongqing, Zhengzhou, China, China

Jin, Weiwei, Beijing, China

Ju, Yoonha, Manhattan, KS, USA

Kaeppler, Heidi, Madison, WI, USA

Kale, Sandip, Gatersleben, Germany

Kamal, Nadia, Neuherberg, Germany

Keller, Beat, Zurich, Switzerland

Kenworthy, Kevin, Gainesville, FL, USA

Kianian, Shahryar, St. Paul, MN, USA

Kong, Xiuying, Beijing, China

Kong, Weilong, Wuhan, China

Krattinger, Simon, Thuwal

Kumar, Satish, Havelock North, New Zealand

Kumar, Rohit, Clemson, SC, USA

Kurdyukov, Sergey, Camperdown, New South Wales, Australia

Lam, Hon‐Ming, Shatin, N.T., Hong Kong

Lei, Li, Berkeley, CA, USA

Leiser, Willmar, Stuttgart, Germany

Li, Xuehui, Fargo, ND, USA

Li, Ying, West Lafayette, IN, USA

Li, Huihui, Beijing, China

Li, Mingjun, Yangling, Shaanxi, China

Li, Pinghua, Tai'an, Shandong, China

Lima, Dayane

Lipka, Alexander, Urbana, IL, USA

Lisch, Damon, West Lafayette, IN, USA

Liu, Bao, Changchun, China

Liu, Hongjun, Chengdu, China

Liu, Sanzhen, Manhattan, KS, USA

Liu, Xiaotong, Minneapolis, MN, USA

Lozano, Roberto, Ithaca, NY, USA

Luo, Ming‐Chen

Ma, Shisong

Mace, Emma, Warwick, Queensland, Australia

MacLean, Dan, Norwich, United Kingdom of Great Britain and Northern Ireland

Madlung, Andreas, Tacoma, WA, USA

Madrid, Eva, Cologne, Germany

Mascher, Martin, Stadt Seeland, Germany

Mastrangelo, Anna Maria, Bergamo, Italy

Meksem, Khalid, Carbondale, IL, USA

Mengiste, Tesfaye, West Lafayette, IN, USA

Micheletti, Diego

Minamikawa, Mai, Tokyo, Japan

Miranda, Carrie, Fargo, ND, USA

Miskovic, Ljubisa, Lausanne, Switzerland

Monat, Cecile, Clermont‐Ferrand, France

Montesinos‐López, Osval, Colima, Mexico

Monteverde, Eliana, Ithaca, NY, USA

Morris, Geoffrey, Manhattan, KS, USA

Mower, Jeffrey, Lincoln, NE, USA

Muthamilarasan, M.

Neik, Ting Xiang (Tina), Bandar Sunway, Selangor, Malaysia

Okut, Hayrettin

Osorio, Luis, Wimauma, FL, USA

Paczos‐Grzeda, Edyta, Lublin, Poland

Paran, Ilan, Bet‐Dagan, Israel

Parkin, Isobel, Saskatoon, Saskatchewan, Canada

Parsons, Josh, Plano, TX, USA

Patil, Gunvant, Lubbock, TX, USA

Pepper, Alan, College Station, TX, USA

Pidon, Hélène, Stadt Seeland, Germany

Poptsova, Maria

Porch, Timothy, Mayaguez, Puerto Rico

Qiao, Pengfei, Ithaca, USA

Qiu, Li‐juan, Beijing, China

Rabanus‐Wallace, Mark, Gatersleben, Germany

Rabanus‐Wallace, Mark Timothy

Ramasamy, Perumal

Ramstein, Guillaume, Ithaca, NY, USA

Rong, Junkang, Hangzhou, China

Rutkoski, Jessica, Urbana, IL, USA

Salvi, Silvio, Bologna, Italy

Sanchez‐Martin, Javier

Sandoya, German

Schmidt, Renate

Schnable, James, Lincoln, USA

Septiningsih, Endang, College Station, TX, USA

Sharma, Sanjeev, Dundee, United Kingdom of Great Britain and Northern Ireland

Sharma, Jyoti, Morden, Manitoba, Canada

Shikanai, Toshiharu

Shrestha, Sandesh, Manhattan, KS, USA

Sonah, Humira

Song, Hui, Qingdao, China

Soriano, Jose

Spannagl, Manuel, Neuherberg, Germany

Sperotto, Raul Antonio

Springer, Nathan, Saint Paul, MI, USA

Steele, K.

Steuernagel, Burkhard, Norwich, United Kingdom of Great Britain and Northern Ireland

Stewart, Phillip

Stich, Benjamin, Dusseldorf, Germany

Stockinger, Eric

Stupar, Robert, Saint Paul, MN, USA

Subramanian, Senthil

Sukumaran, Sivakumar, Texcoco, Mexico, Mexico

Sun, Xiaoqin, Nanjing, Jiangsu, China

Teh, Soon Li, Wenatchee, WA, USA

Thomson, Michael, College Station, TX, USA

Thyssen, Gregory, New Orleans, Louisiana, USA

Tilley, Mike

Toth, Jacob, Ithaca, NY, USA

Tran, Lam‐Son

tripodi, pasquale, Pontecagnano, Italy

Tuberosa, Roberto, Bologna, Italy

Vail, Sally

Valliyodan, Babu

van Dijk, Thijs, Eck en Wiel, Netherlands

Vandepoele, Klaas

Vega, Juan, Madrid, Madrid, Spain

Villarino, Gonzalo H., USA

Vining, Kelly, Corvallis, OR, USA

Vuong, Tri, Columbia, MO, USA

Walkowiak, Sean

Wang, Guan‐Feng, Qingdao, China

Wang, Ming Li, Griffin, GA, USA

Wang, Xutong

Washburn, Jacob

Weber, Gerd, Stuttgart, Germany

Wen, Feng, Jiujiang, China

Weng, Yiqun, Madison, WI USA

Werner, Christian, Giessen, Germany

Westbrook, Jared, Asheville, NC, USA

Widrlechner, Mark, Ames, IA, USA

Wingen, Luzie, Norwich, United Kingdom of Great Britain and Northern Ireland

Wu, Shawn

Xia, Xianchun, Beujing, China

Xing, Libo

Xiong, Lizhong, Wuhan, China

Xu, Pei

Xu, Steven, Fargo, ND, USA

Yadegari, Ramin

Ye, Chu‐Yu, Hangzhou, China

Yu, Mingliang, Nanjing, China

Zhang, Bo

Zhang, Liangsheng

Zhang, Qiong

Zhang, Yuanyuan, Wuhan, Hubei, China

Zhang, Peng, Hangzhou, China

Zhang, Feng, USA

Zhang, Jun

Zhao, Yanxin, Beijing, China

Zhao, Yunpeng, Hangzhou, Zhejiang, China

Zhao, Yusheng, Stadt Seeland, Germany

Zheng, Chuanlin, Beijing, China

Zheng, Xiaoming, Beijing, China

Zhong, Yan, Nanjing, China

Zhou, Peng, St. Paul, MN, USA

Zou, Jun, Wuhan, China

